# Supplementary material for: Periaqueductal gray activates antipredatory neural responses in the amygdala of foraging rats
Source: eLife. 2024 Aug 12;12:RP88733. doi: 10.7554/eLife.88733 (PMC11318971; doi:10.7554/eLife.88733)
Supplement: Supplementary file 1. — (A) Histological reconstructions of recording sites in the dPAG and BLA, and optic fiber locations in the dPAG. (A) Red bars show the trajectory of tetrode recording sites in the dPAG. (B) Red bars depict the trajectory of optrode recording sites in the dPAG. (B) Red and gray circles represent the optic fiber locations for ChR2 and EYFP rats, respectively. (D) Orange circles and red bars indicate the optic fiber locations in the dPAG (left) and recording trajectories in the BLA (right), respectively. Numerical values represent AP coordinates relative to Bregma. (B) Normality test. The normality of the variable distributions was assessed using the Kolmogorov–Smirnov test (p<0.01). Depending on the results of this test, parametric tests were used for normally distributed variables, while nonparametric tests were employed for variables that were not normally distributed. [file elife-88733-supp1.docx]

**Supplementary File 1A**

**
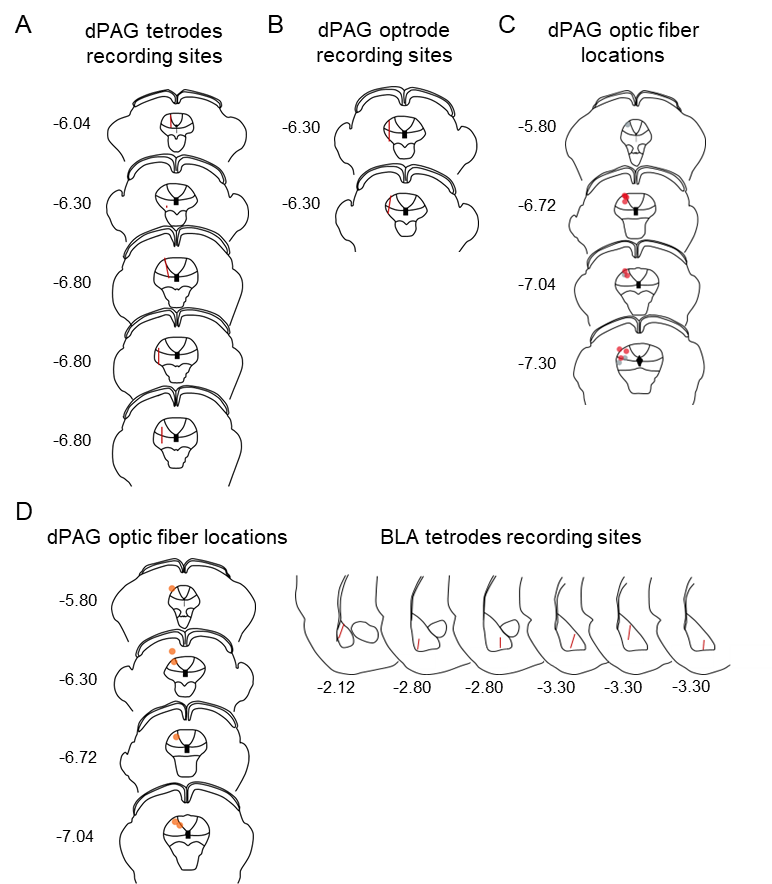
**

**Supplementary File 1B**

| Fig | Variables | KS distance | P | Passed  (α = .01)? | Fig | Variables | KS distance | P | Passed  (α = .01)? |
| --- | --- | --- | --- | --- | --- | --- | --- | --- | --- |
| 1C | Outbound foraging time (s) | | | | 4H | CTB density (% of control) | | | |
|  | pre | 0.2916 | <0.0001 | No |  | Foraging only | 0.2191 | >0.1000 | Yes |
|  | robot | 0.1713 | 0.0033 | No |  | Robot experienced | 0.3914 | 0.0118 | Yes |
|  | post | 0.2807 | <0.0001 | No |  | (cFos+CTB)/CTB (% of control) | | | |
| 1D | Success rate (%) | | | |  | Foraging only | 0.267 | >0.1000 | Yes |
|  | pre | 1 | <0.0001 | No |  | Robot experienced | 0.1972 | >0.1000 | Yes |
|  | robot | 0.5102 | <0.0001 | No | S1D | Average firing (spikes/s) | | | |
|  | post | 1 | <0.0001 | No |  | Pre-robot | 0.3185 | <0.0001 | No |
| 1F | PAG robot cells firing (Z) | | | |  | Robot | 0.3045 | <0.0001 | No |
|  | pre_bin1 (0-1 s) | 0.1094 | >0.1000 | No |  | Post-robot | 0.3197 | <0.0001 | No |
|  | pre_bin2 (1-2 s) | 0.2252 | 0.005 | No |  | Max firing (spikes/s) | | | |
|  | pre_bin3 (2-3 s) | 0.2933 | <0.0001 | No |  | Pre-robot | 0.2578 | 0.0005 | No |
|  | pre_bin4 (3-4 s) | 0.2553 | 0.0006 | No |  | Robot | 0.1969 | 0.0263 | Yes |
|  | pre_bin5 (4-5 s) | 0.2324 | 0.0031 | No |  | Post-robot | 0.2103 | 0.0125 | Yes |
|  | robot_bin1 (0-1 s) | 0.2035 | 0.0184 | Yes | S3H | AUC (Z, 0-0.5s) | | | |
|  | robot_bin2 (1-2 s) | 0.2038 | 0.0181 | Yes |  | Stim cells | 0.2519 | <0.0001 | No |
|  | robot_bin3 (2-3 s) | 0.1826 | 0.0544 | Yes |  | nonStim cells | 0.1462 | >0.1000 | Yes |
|  | robot_bin4 (3-4 s) | 0.1913 | 0.0353 | Yes | S3I | AUC (Z, 0-5s) | | | |
|  | robot_bin5 (4-5 s) | 0.1564 | >0.1000 | Yes |  | Stim cells | 0.2161 | <0.0001 | No |
|  | post_bin1 (0-1 s) | 0.1948 | 0.0294 | Yes |  | nonStim cells | 0.1709 | 0.0799 | Yes |
|  | post_bin2 (1-2 s) | 0.153 | >0.1000 | Yes | S4B | CC AUC (Z) | | | |
|  | post_bin3 (2-3 s) | 0.0917 | >0.1000 | Yes |  | pre | 0.2343 | 0.0008 | No |
|  | post_bin4 (3-4 s) | 0.1499 | >0.1000 | Yes |  | stim | 0.2587 | <0.0001 | No |
|  | post_bin5 (4-5 s) | 0.1822 | 0.0556 | Yes |  | post | 0.2898 | <0.0001 | No |
| 2G-J | Control group n (=4) was too small to analyze normality.  Nonparametric statistics were used for analyzing the data. | | | |  | robot | 0.1273 | >0.1000 | Yes |
|  |  |  |  |  | S4C | CC peak (Z) | | | |
| 3C | Outbound foraging time (s) | | | |  | pre | 0.1998 | 0.009 | No |
|  | pre | 0.1481 | 0.0002 | No |  | stim | 0.2359 | 0.0007 | No |
|  | robot | 0.1155 | 0.0119 | Yes |  | post | 0.3407 | <0.0001 | No |
|  | post | 0.1483 | 0.0002 | No |  | robot | 0.1926 | 0.0142 | Yes |
| 3D | Success rate (%) | | | | S4E | CC AUC (Z) | | | |
|  | pre | 1 | <0.0001 | No |  | pre | 0.2408 | 0.0138 | Yes |
|  | robot | 1 | <0.0001 | No |  | stim | 0.2842 | 0.0012 | No |
|  | post | 1 | <0.0001 | No |  | post | 0.1278 | >0.1000 | Yes |
| 3G | (Outbound foraging time (s) | | | |  | robot | 0.2174 | 0.0417 | Yes |
|  | pre | 0.1375 | >0.1000 | Yes |  | CC peak (Z) | | | |
|  | stim | 0.1758 | >0.1000 | Yes |  | pre | 0.2297 | 0.0237 | Yes |
|  | post | 0.2018 | 0.0808 | Yes |  | stim | 0.2183 | 0.0401 | Yes |
|  | robot | 0.2389 | 0.0152 | Yes |  | post | 0.3022 | 0.0004 | No |
| 3M | Relative firings (Z) - All significant pairs | | | |  | robot | 0.2014 | 0.0821 | Yes |
|  | pre | 0.1998 | 0.009 | No | S4F | CC AUC (Z) | | | |
|  | stim | 0.2359 | 0.0007 | No |  | pre | 0.2647 | 0.0454 | Yes |
|  | post | 0.3407 | <0.0001 | No |  | stim | 0.2759 | 0.0298 | Yes |
|  | robot | 0.1926 | 0.0142 | Yes |  | post | 0.3317 | 0.0026 | No |
| 3N | Relative firings (Z) - Stim pairs | | | |  | robot | 0.1438 | >0.1000 | Yes |
|  | pre | 0.2297 | 0.0237 | Yes |  | CC peak (Z) | | | |
|  | stim | 0.2183 | 0.0401 | Yes |  | pre | 0.2153 | >0.1000 | Yes |
|  | post | 0.3022 | 0.0004 | No |  | stim | 0.2321 | >0.1000 | Yes |
|  | robot | 0.2014 | 0.0821 | Yes |  | post | 0.4233 | <0.0001 | No |
|  | Relative firings (Z) - nonStim pairs | | | |  | robot | 0.3327 | 0.0025 | No |
|  | pre | 0.2153 | >0.1000 | Yes | S4G | CC AUC (Z) | | | |
|  | stim | 0.2321 | >0.1000 | Yes |  | stim pair (0-20 ms) | 0.1021 | >0.1000 | Yes |
|  | post | 0.4233 | <0.0001 | No |  | stim pair (20-40 ms) | 0.2242 | 0.0307 | Yes |
|  | robot | 0.3327 | 0.0025 | No |  | stim pair (40-60 ms | 0.192 | >0.1000 | Yes |
| 4C | Latency to procure pellets (s) | | | |  | stim pair (60-80 ms) | 0.2829 | 0.0013 | No |
|  | Base_Foraging only | 0.3539 | 0.0181 | Yes |  | stim pair (80-100 ms) | 0.2552 | 0.0065 | No |
|  | Base_Robot experienced | 0.2801 | 0.0253 | Yes |  | nonstim pair (0-20 ms) | 0.1589 | >0.1000 | Yes |
|  | Test_Foraging only | 0.2047 | >0.1000 | Yes |  | nonstim pair (20-40 ms) | 0.2269 | >0.1000 | Yes |
|  | Test_Robot experienced | 1 | <0.0001 | No |  | nonstim pair (40-60 ms) | 0.2308 | >0.1000 | Yes |
| 4E | Fos-positive cells (% of control) | | | |  | nonstim pair (60-80 ms) | 0.3189 | 0.0048 | No |
|  | PVT_Foraging only | 0.2505 | >0.1000 | Yes |  | nonstim pair (80-100 ms) | 0.2742 | 0.0319 | Yes |
|  | PVT_Robot experienced | 0.3508 | 0.0010 | No | S4H | CC peak | | | |
|  | IMD_Foraging only | 0.2808 | >0.1000 | Yes |  | Stim pairs | 0.3327 | 0.0025 | No |
|  | IMD_Robot experienced | 0.1831 | >0.1000 | Yes |  | nonStim pairs | 0.2014 | 0.0821 | Yes |
|  | CM_Foraging only | 0.2105 | >0.1000 | Yes | S5 | CTB density (cells/0.1 mm^2^) | | | |
|  | CM_Robot experienced | 0.2463 | 0.0866 | Yes |  | Foraging only | 0.2488 | >0.1000 | Yes |
|  | Rh_Foraging only | 0.2892 | >0.1000 | Yes |  | Robot experienced | 0.3321 | 0.0748 | Yes |
|  | Rh_Robot experienced | 0.1480 | >0.1000 | Yes |  | % (cFos+CTB)/CTB | | | |
|  | Re_Foraging only | 0.1952 | >0.1000 | Yes |  | Foraging only | 0.2206 | >0.1000 | Yes |
|  | Re_Robot experienced | 0.1330 | >0.1000 | Yes |  | Robot experienced | 0.2192 | >0.1000 | Yes |
